# Supplementary material for: Rapid fabrication of vascularized and innervated cell-laden bone models with biomimetic intrafibrillar collagen mineralization
Source: Nat Commun. 2019 Aug 6;10:3520. doi: 10.1038/s41467-019-11455-8 (PMC6684598; doi:10.1038/s41467-019-11455-8)
Supplement: Supplementary file 1 — Supplementary Information [file 41467_2019_11455_MOESM1_ESM.pdf]

**Supplementary Information**

Rapid fabrication of vascularized and innervated cell-laden bone models with biomimetic intrafibrillar collagen mineralization

Thrivikraman et al.

## Supplementary Figures

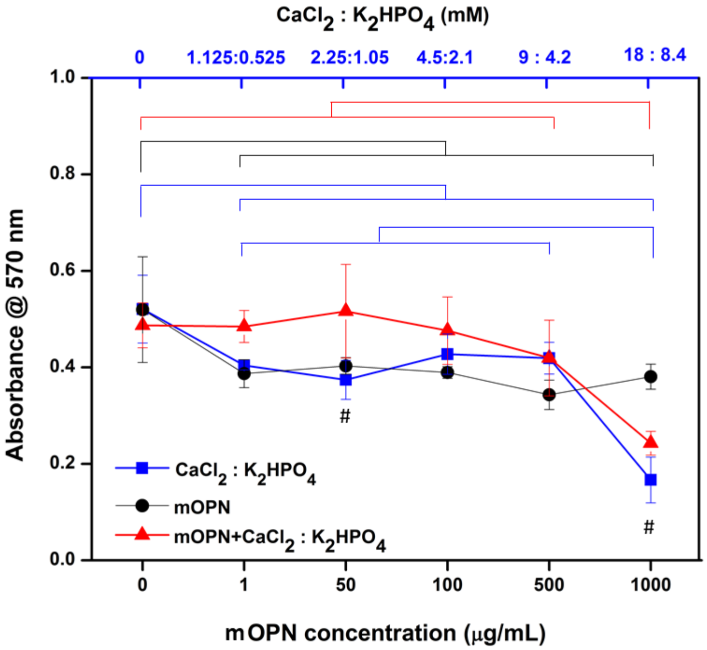


**Supplementary Figure 1:** Effect of mineralizing medium on cell cytotoxicity. Cytotoxicity was determined using an MTT (3-(4,5-Dimethylthiazol-2-yl)-2,5-Diphenyltetrazolium Bromide) assay kit on an adherent monolayer of hMSCs exposed to mOPN alone, and varying concentrations of calcium and phosphate-supplemented medium, with or without mOPN as a mineralization process-directing agent. Untreated cells cultured on tissue-culture plastic were used as a control. Briefly, hMSCs were seeded in a 96 well plate at a density of $5X10^3$ cells/well and the following day, cells were treated. After 48 hrs of incubation, the MTT reagent was added and the samples were incubated at 37°C for 4 hours. The resulting purple colored formazan crystals were dissolved in DMSO and the absorbance was measured spectrophotometrically at 570 nm using a microplate reader. Exposure to increasing concentrations of calcium and phosphate containing medium resulted in a significant reduction in cell viability. In contrast, treatment with mOPN alone did not affect the cell viability, except for a slight reduction. Interestingly, the concentration dependent cytotoxicity response of calcium and phosphate mineralizing medium was significantly mitigated in the presence of mOPN, even at the highest concentration tested. Such a negated cytotoxicity effect of calcium and phosphate when used in combination with mOPN may possibly be due to the formation of amorphous calcium phosphate precursors in the culture medium which reduce free CaP ion-induced cellular damage by preventing its intracellular uptake. Data presented as Mean ± SD. Data points connected by similar color bars represent a significant difference within the groups ($p<0.05$, ANOVA/Tukey), whereas data points underlined with # indicate a significant difference between groups. (N=4). Source data are provided as a Source Data file.

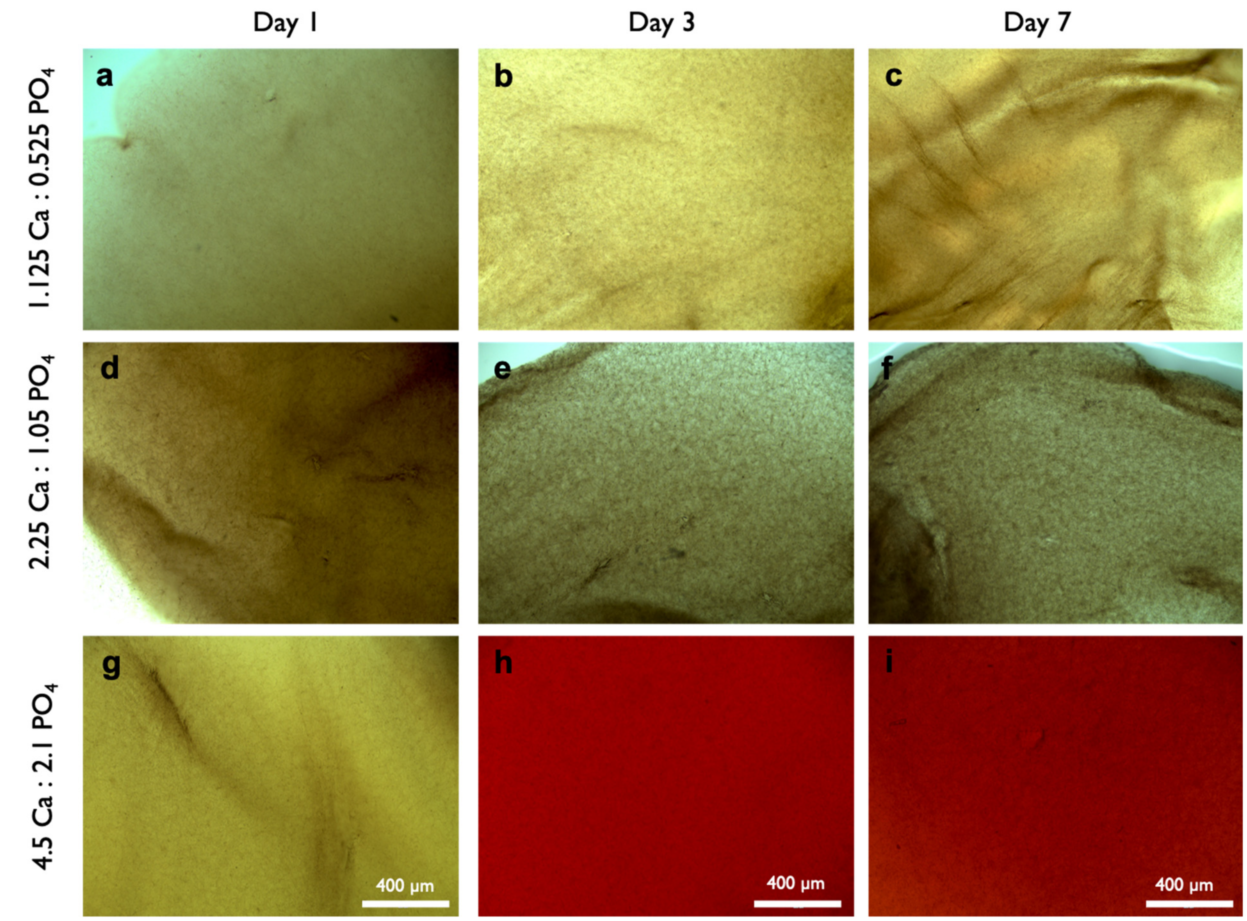


**Supplementary Figure 2:** Alizarin red staining for collagen hydrogels mineralized with increasing concentrations of $Ca^{2+}$ and $PO_4^{3-}$. Samples were treated with 1.125 mM $Ca^{2+}$ and 0.525 mM $PO_4^{3-}$ daily for 3 days and were analyzed on days (a) 1, (b) 3 and (c) 7. Virtually no red staining is present even after 7 days. The same pattern of negligible mineralization is seen for samples treated with 2.25 mM $Ca^{2+}$ and 1.05 mM $PO_4^{3-}$ (d-f). For samples cultured using (g) 4.5 mM $Ca^{2+}$ and 2.1 mM $PO_4^{3-}$, a marked increase in Alizarin Red staining is seen on (h) day 3, and the red hue is maintained up to day 7 (i) (N=6).

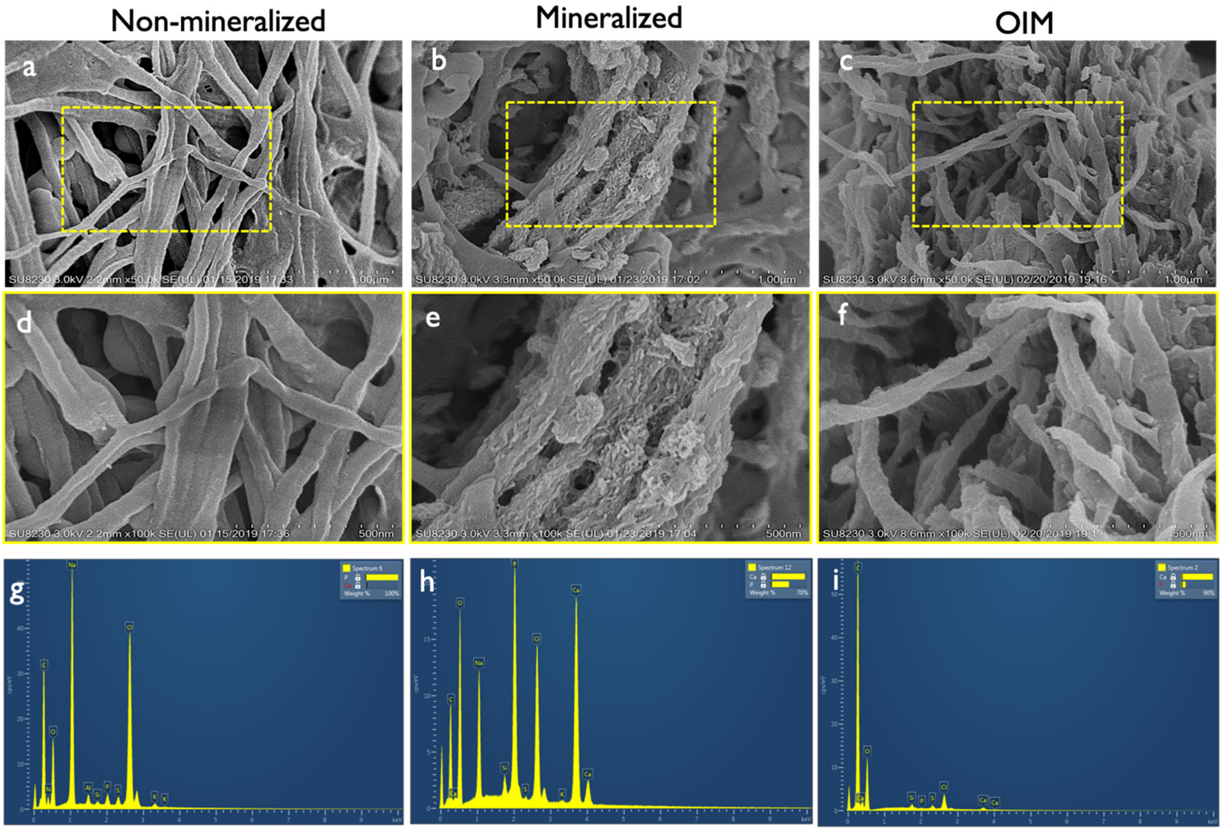


**Supplementary Figure 3:** SEM micrographs showing representative (a, d) non-mineralized, (b, e) mineralized and (c, f) in OIM-treated collagen hydrogels after 3 days in lower and higher magnification. (g-i) show the respective elemental composition of each group, where both non-mineralized (g) and OIM-treated samples (i) had only minor Ca and P peaks, while high peaks for both Ca and P were present in the mineralized samples (h). (N=4)

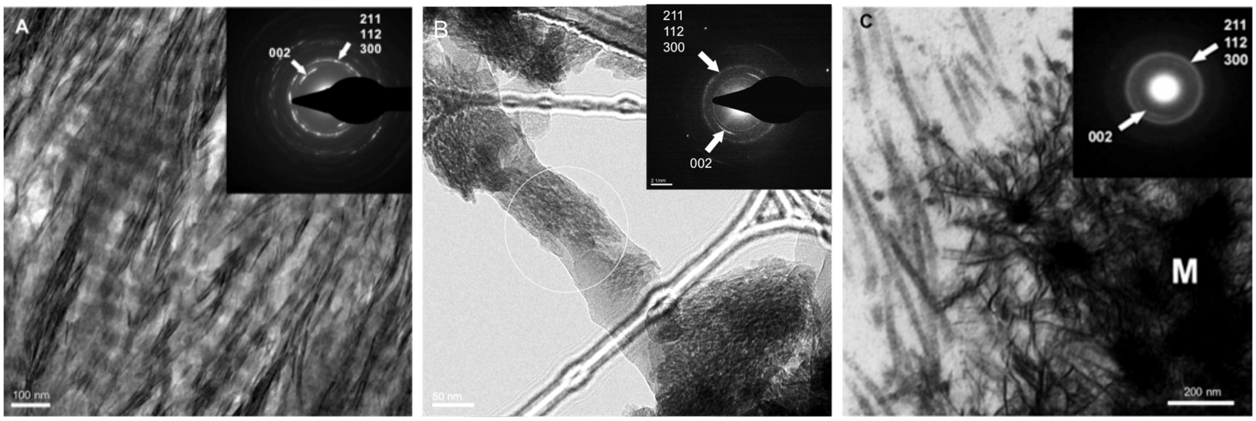


**Supplementary Figure 4:** Selective Area Electron Diffraction (SAED) analyses of (a) native bone, (b) mineralized collagen hydrogels after 3 days, and (c) osteoblast-secreted mineralized matrix [Adapted from Boonrungsiman et al.[35] with permission]. Despite the different intensities, the typical (002) plane and overlapping arcs for the (112), (211), and (300) are seen in all three samples, which is suggestive of the crystalline nature of the hydroxyapatite in all systems.

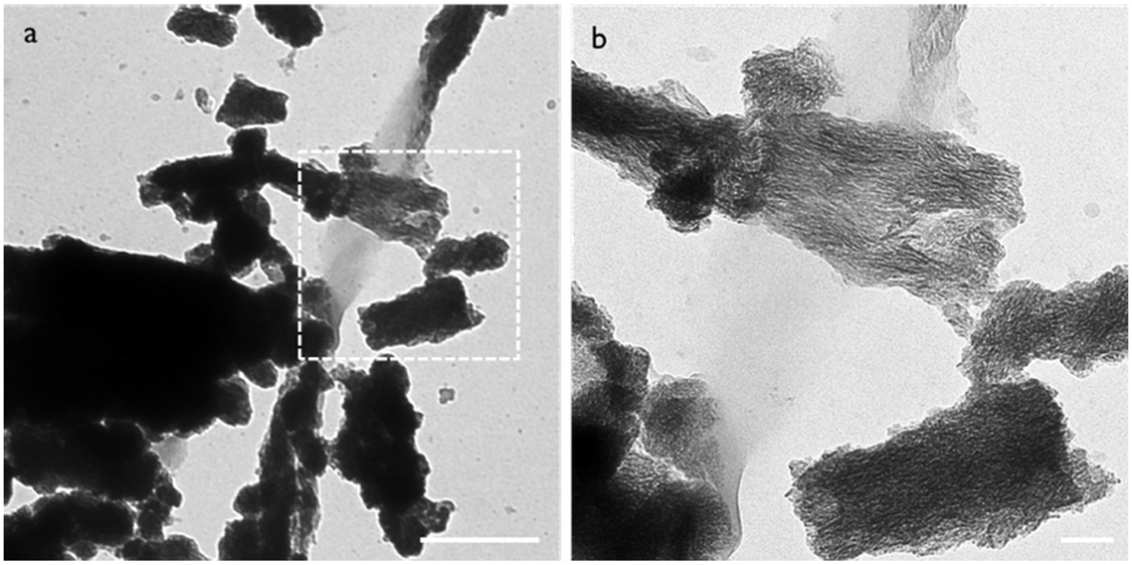


**Supplementary Figure 5:** (a) TEM image of mineralized collagen (unstained) lyophilized and pulverized in liquid nitrogen. Scale bar: 500 nm (b) Needle shaped apatite crystals occupying both the intrafibrillar and extrafibrillar space of collagen fibrils can be visualized in the high magnification image (from the white dotted square in a). Note that the continuity of crystal orientation along the long axis of the fibers is disrupted primarily during TEM sample processing that involved crushing and dispersing the collagen fibrils on TEM grids, resulting in short and randomly oriented fibers/crystallites. A segment of the fibril that did not mineralized (faint contrast) is also present. Scale bar: 100 nm (N=3)

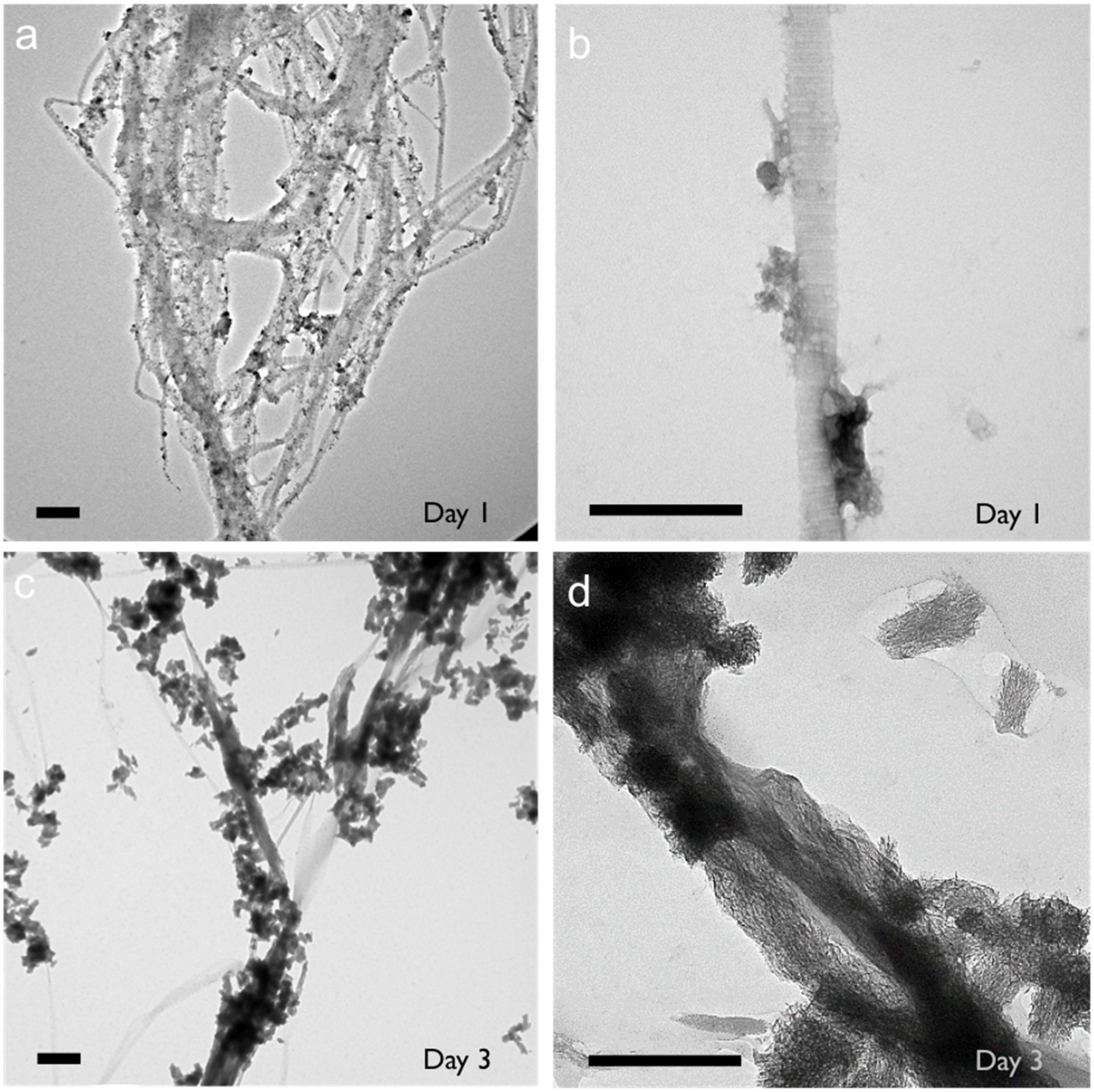


**Supplementary Figure 6:** TEM images showing the progressive mineralization of 3D collagenous matrix from day 1 to day 3. (a) Unstained TEM image depicting partially mineralized fibrils at day 1, with numerous mineral clusters deposited onto the surface of collagen fibrils. The corresponding high magnification image is shown in b. Scale bar: 500 nm (c,d) After 3 days of mineralization, extensive needle-like apatite deposition was observed, both at the intra and extrafibrillar space. Scale bar: 250 nm (N=3)

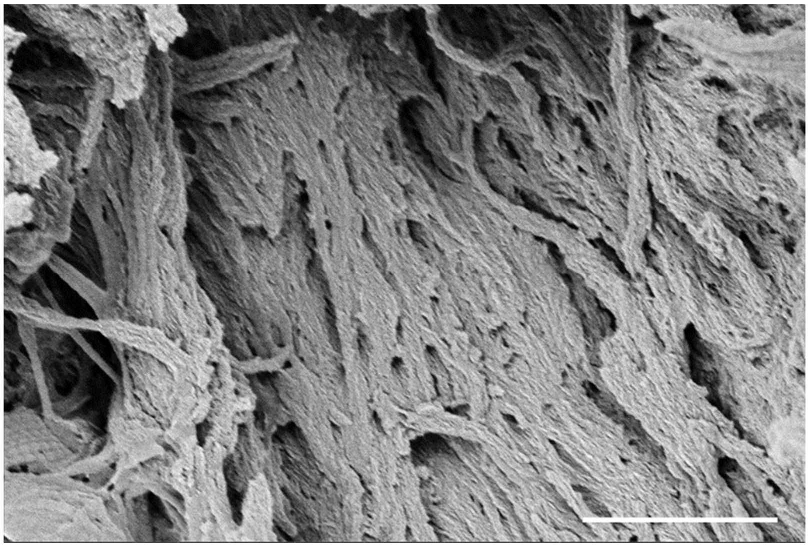

**Supplementary Figure 7:** SEM image showing the mineralized collagenous matrix of human bone. The absence of characteristic D-periodicity in collagen fibrils suggests mineral deposition within the intrafibrillar space. The presence of extrafibrillar mineral surrounding and encasing the densely packed fibril bundles is also noticeable. Scale bar: 1 µm. (N=3)

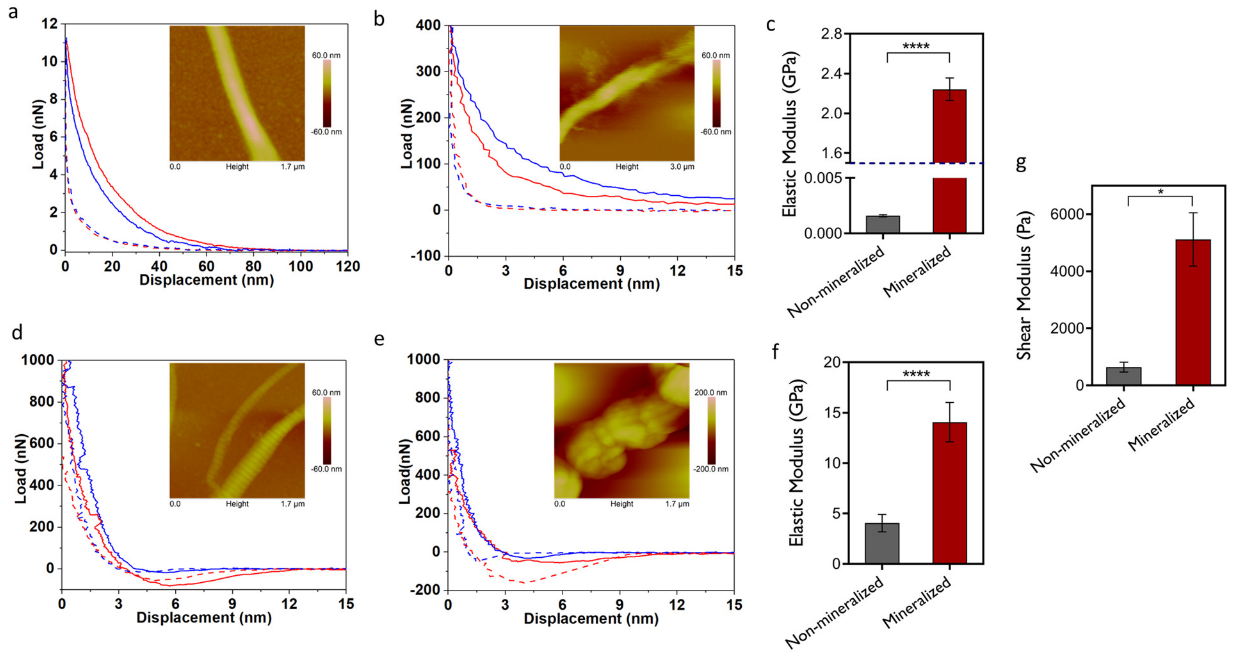


**Supplementary Figure 8:** Analysis of nanoindentation elastic modulus of non-mineralized and mineralized collagen fibrils performed in water (a-c) and in air (d-f) using an atomic force microscope (Nanoscope 8 atomic force microscope, J scanner, Bruker). Representative load-displacement curves on (a) non-mineralized and (b) mineralized collagen fibrils, measured in water. The loading curve is represented in red and the unloading curve is represented in blue. The dotted line corresponds to the indentation on fibrils, while the indentation on the adjacent mica substrate is denoted by the solid line. Inset shows the representative AFM topographic image of individual fibrils fixed onto freshly cleaved muscovite mica disc, with the color scale on the right corresponding to the height. (c) Comparison of average elastic modulus in non-mineralized versus mineralized fibrils, calculated by fitting the Hertz model to the loading curves. An increase of over 1000-fold was recorded in hydrated mineralized fibrils versus non-mineralized controls (****$p<0.0001$, Student's t-test). Corresponding load-displacement curves and average modulus of non-mineralized and mineralized fibrils in air are shown in (d), (e) and (f), respectively. (f) The nanoscale elastic moduli values in air followed the same trend, with a substantial increase in stiffness in mineralized fibrils compared to its non-mineralized counterparts (****$p<0.0001$, Student's t-test). In all cases, the maximum penetration depth was set to ~ 15% of the fibril thickness. In (b-e) the measurements were made using aluminum-coated, silicon microsphere tip of radius, 10 nm, resonance frequency of 300 kHz and a nominal spring constant of 26 N/m whereas for non-mineralized collagen fibril in (a), indentation was performed using triangular gold-coated silicon nitride tip of 30 nm radius, 65 kHz resonance frequency and 0.35 N/m nominal spring constant. Measurements were performed on 5 to 10 different locations from at least 3 fibrils per sample, with a total of 3 samples per condition. (g) The bulk elasticity (shear storage moduli) of mineralized hydrogels was significantly higher compared to their

non-mineralized controls (*p<0.05, Student's t-test). An oscillatory rheometer fitted with an 8 mm parallel plate geometry was used to measure the storage modulus and loss modulus in frequency sweep mode at 1 Hz frequency and 1% strain (N=4). Source data are provided as a Source Data file.

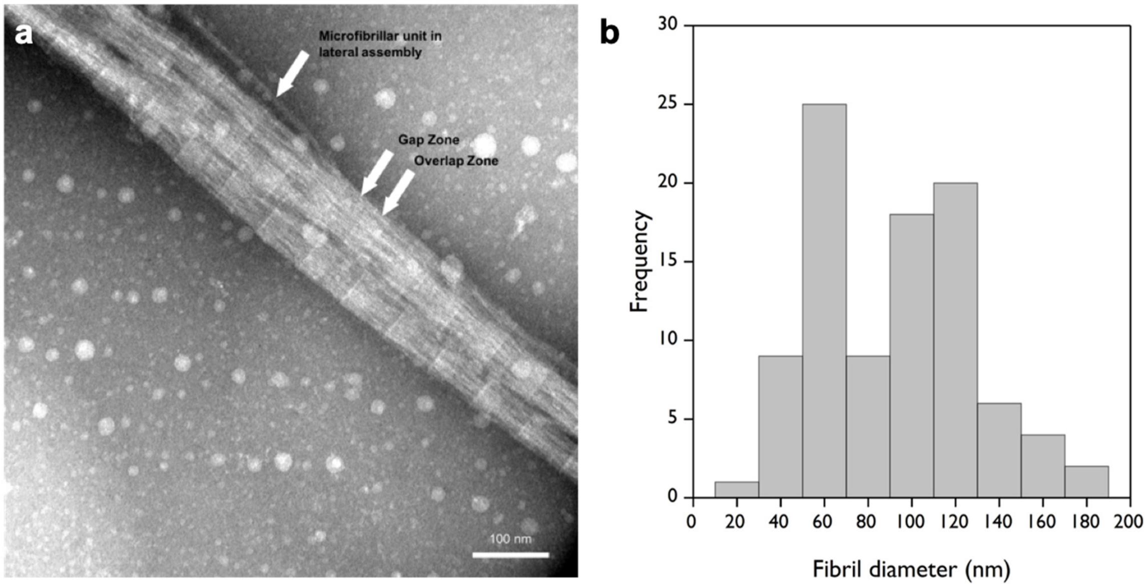


**Supplementary Figure 9:** a) TEM image illustrating the alignment and banding pattern of collagen fibrils used for cell and mineralization studies. b) Frequency plot of fibril diameter from a total of 96 measurements (N=4). The fibrils diameter was typically in the range of 60-120 nm, with higher frequencies found for 60, 100 and 120 nm diameter fibrils. Source data are provided as a Source Data file.

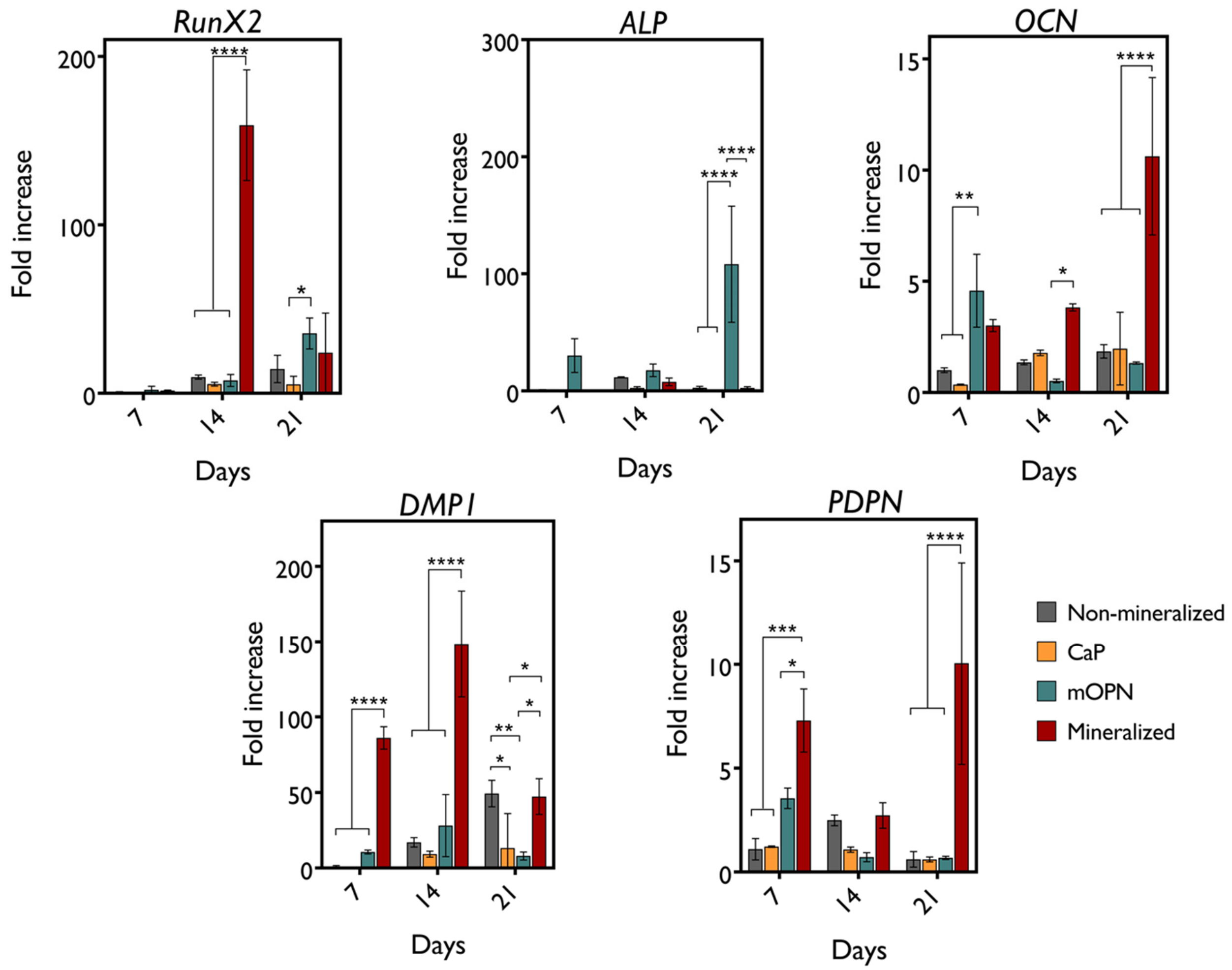


**Supplementary Figure 10:** Gene expression analyses for hMSCs in cell-laden hydrogels after 7, 14 and 21 days of culture, with and without matrix mineralization, and in comparison to cells cultured in medium supplemented with calcium and phosphate, or mOPN alone. The culture of hMSCs in CaP-containing medium alone did not induce a significant upregulation of osteogenic markers, while incubation in mOPN alone had a significant increase in mRNA levels for OCN at day 7 (**p<0.01) and ALP at D21 (****p<0.0001). One would generally expect a significant upregulation in ALP production during early stages of osteoblastic differentiation, and a subsequent drop as cells mature into osteocytes. However, in our mineralized samples a reduction in ALP expression level was recorded from the earliest time point.  Functionally, ALP stimulates mineral deposition by hydrolyzing inorganic pyrophosphate to liberate inorganic phosphate (Pi), and studies discussed in the main text in more detail suggest that a high extracellular Pi content can in turn inhibit ALP activity via a negative feedback mechanism, which is in agreement with our findings. Data are represented as Mean±SD (N=3). Source data are provided as a Source Data file.

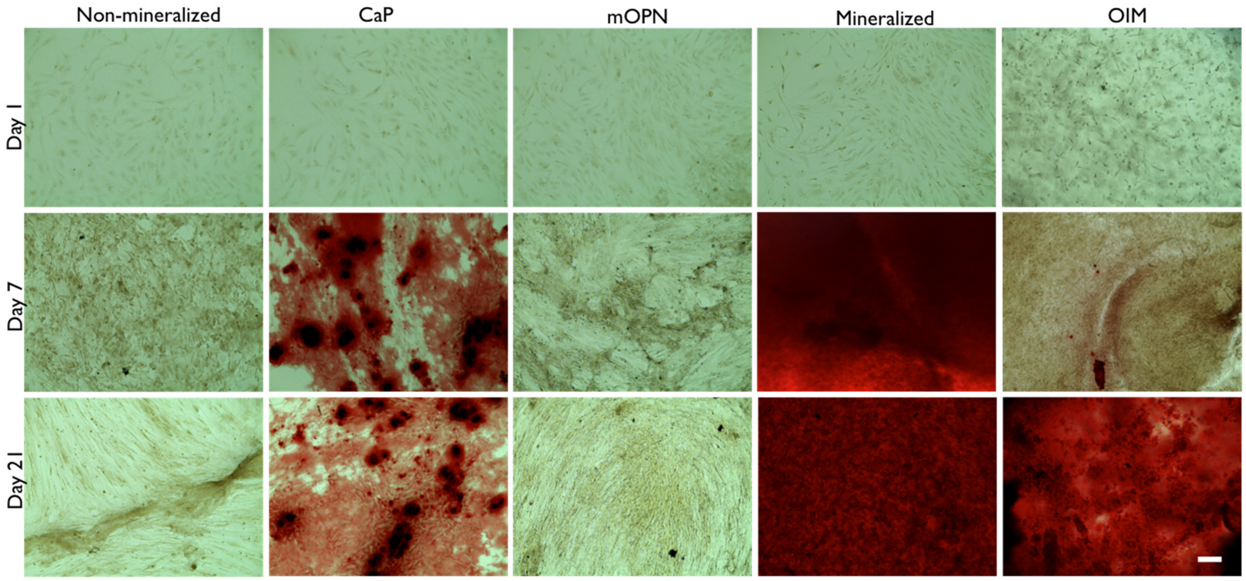


**Supplementary Figure 11:** Representative alizarin red staining images depicting homogenous calcification in the mineralized constructs within 7 days in culture. A total of 5 X $10^4$ cells were encapsulated in collagen (1.5 mg/ml) hydrogels (N=4). For the CaP condition, cells were cultured in DMEM supplemented with 4.5 mM of $CaCl_2$ and 2.1 mM of $K_2HPO_4$. For the mOPN condition, DMEM supplemented with 100 μg/mL of mOPN was used; In OIM group, the cells were cultured in DMEM containing 100 nM Dexamethasone, 50 μM ascorbic acid and 10 mM β-glycerol phosphate. Non-mineralized constructs were cultured in non-supplemented complete DMEM medium. After 1, 7 and 21 of culture, Alizarin Red S (Sigma) staining was performed to assess the overall mineralization. In brief, after culture, samples were washed in PBS, fixed in 4% paraformaldehyde for 5 min and then stained using 2% (w/v) Alizarin red solution at pH 4.2 for 5 minutes. After repeated washing in distilled water to remove any unbound stain, the constructs were imaged in bright field mode. Non-mineralized constructs showed no signs of mineral deposition even after 21 days, whereas the constructs exposed to osteoinductive supplements showed mineral nodule formation after 21 days of culture. The constructs treated with CaP containing medium resulted in the random deposition of mineral nodules, while treatment with mOPN alone did not induce any visible mineral deposition. Of note, intense red staining was first detected in the mineralized construct at day 3 (Supplementary Figure 2), maintained at day 7 and day 21, suggesting the presence of dense calcium phosphate deposits uniformly distributed throughout the matrix. Scale bar: 100 μm

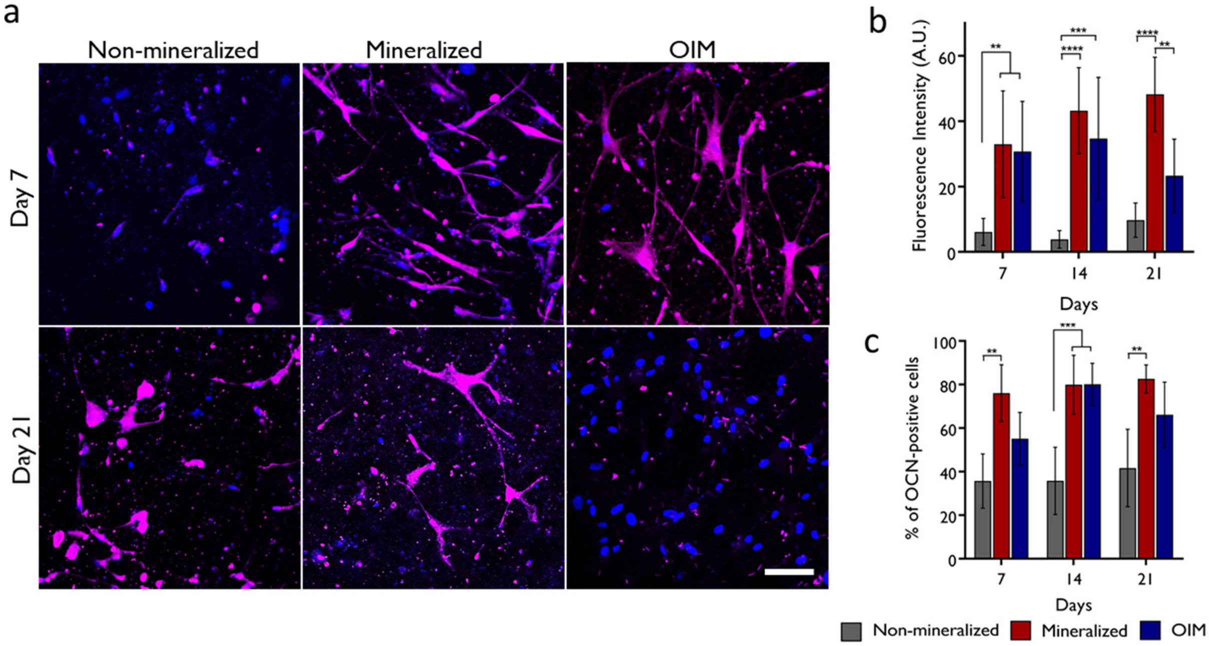


**Supplementary Figure 12:** Expression of osteocalcin in hMSCs encapsulated within non-mineralized versus mineralized hydrogels, as detected by immunofluorescence staining (a). Cells treated with osteoinductive medium served as the positive control. In non-mineralized constructs, OCN expression was minimally detected at the early time point. However, prolonged culture for 21 days within the construct resulted in a slight increase in the OCN expression level. OIM and mineralized constructs, on the other hand readily exhibited intense OCN expression, about 6-fold higher than those in non-mineralized constructs (b), within 7 days of culture and almost 80% differentiation by 14 days (c). Unlike mineralized constructs, that retained OCN expression level after 21 days, cells in OIM had a markedly reduced expression. (*p<0.05; **p<0.01, *p<0.001; ****p<0.0001, by ANOVA test; Data represented as mean±SD; (N=3); Scale bar: 100 μm). Source data are provided as a Source Data file.

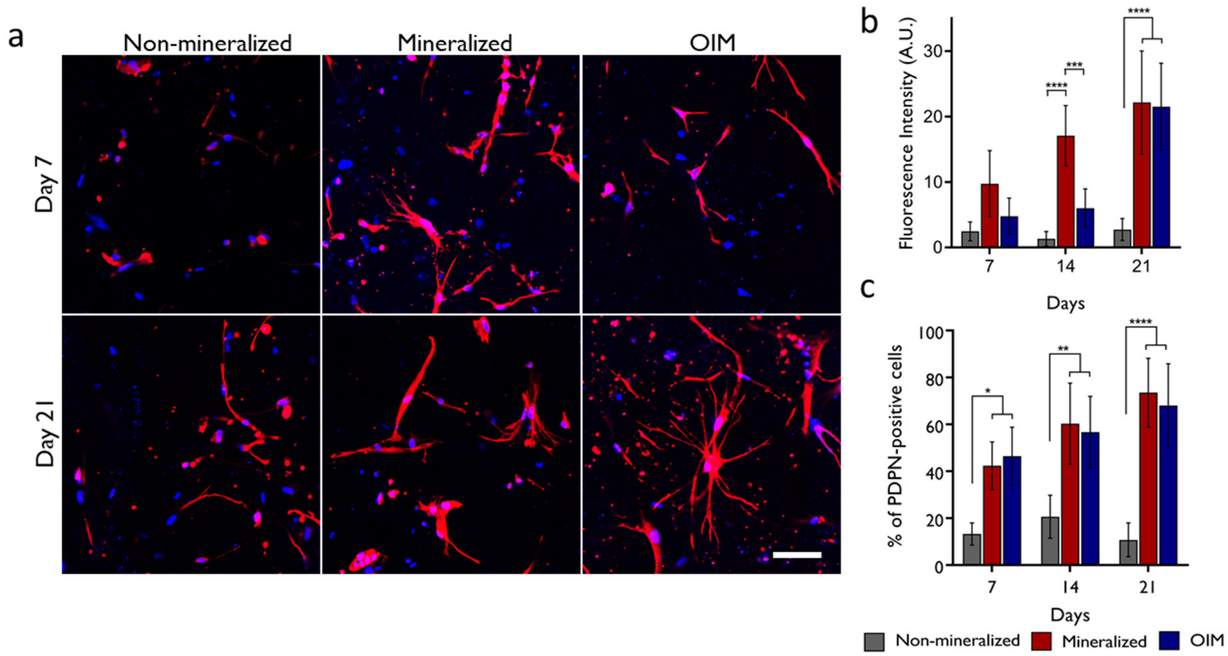


**Supplementary Figure 13:** a) Detection of pre-osteocytic PDPN marker expression in hMSCs by immunofluorescence staining. A time dependent increase in the expression of PDPN was noticed in non-mineralized and positive control groups, whereas the cells in the mineralized constructs showed a high PDPN expression even at the early time point of day 7 (b) with 70% differentiation by day 21 (c). (*p<0.05; **p<0.01, *p<0.001; ****p<0.0001, by ANOVA test; Data represented as mean±SD; (N=3); Scale bar: 100 µm). Source data are provided as a Source Data file.

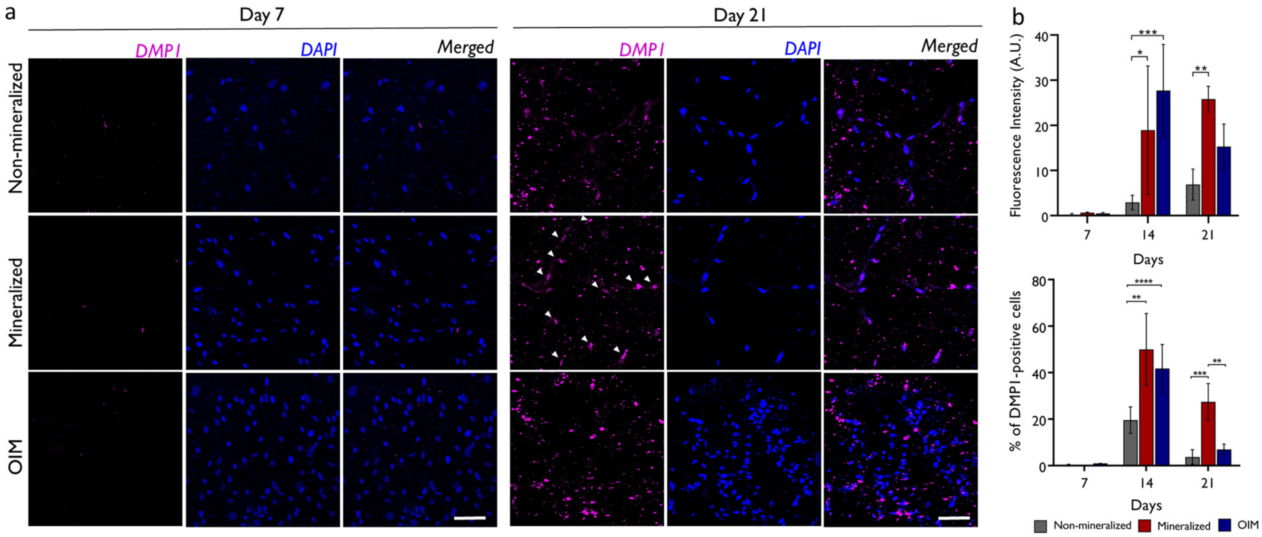


**Supplementary Figure 14**: (a) Expression of pre-osteocytic marker, DMP1 in hMSCs encapsulated within non-mineralized, mineralized and OIM constructs. Cells were immunolabelled with mouse anti-DMP1 antibody, followed by staining with secondary fluorochrome, AlexaFluor 647-conjugated goat anti-mouse IgG antibody. No DMP1 expression was detected in hMSCs at an early time-point of day 7, irrespective of the conditions tested. Conversely, the majority of the nuclei were stained positive for DMP1 in mineralized constructs on day 21 (white arrows) compared to faint nuclear staining in the non-mineralized group. Note the minimal expression of DMP1 in OIM treated group. Non-specific background labeling was noticed in all three constructs after extended culture for 21 days. DAPI is stained in blue. (b) The mean fluorescent intensity was not statistically different between the mineralized and OIM constructs, both on day 14 and 21. (c) Quantification of the percentage of cells expressing DMP1 after 7, 14 and 21 days. The highest fraction of DMP1-positive cells was recorded in the mineralized groups at day 14 and 21, with a statistically significant difference with respect to OIM at day 21. (*$p<0.05$; **$p<0.01$, *$p<0.001$; ****$p<0.0001$, by ANOVA test; Data represented as mean±SD; (N=3); Scale bar: 100 μm). Source data are provided as a Source Data file.

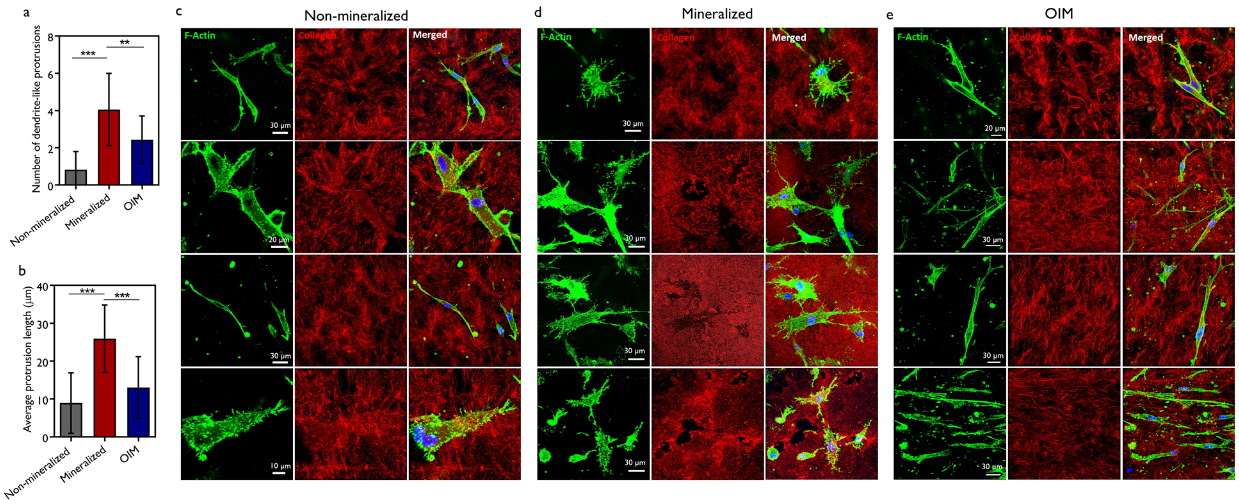


**Supplementary Figure 15:** Quantification of the number (a) and length (b) of dendrite-like protrusions in hMSCs after 7 days of culture in 3D matrices. The cells were fixed, stained for F-actin and imaged using confocal microscopy (63x objective, Zeiss 210 Airyscan LSM 880). The mean number of protrusions per cell was counted manually using the cell counter plugin in imageJ software. The average protrusion length was assessed by manually tracing the extensions with the freehand selection tool and their lengths were measured with ImageJ. Thin filopodial structures of length and width less than 5 µm and 1 µm, respectively were excluded from the data analysis for dendrite-like protrusions. Cells in the mineralized group exhibited a significant increase in the number and length of protrusions compared to non-mineralized and OIM groups. Data represented as Mean±SD, from four independent experiments with at least 30 cells per group. Comparison by one-way Anova (**p<0.01,***p<0.001). Representative confocal images of cells from 4 different biological replicates exhibiting dendrite-like protrusions in 3D non-mineralized (c), mineralized (d) and OIM (e) constructs after 7 days. Small extensions stemming from the protrusions can be particularly noted in cells encapsulated within the mineralized matrix which may play a role in orienting dendrite formation and growth[60]. Source data are provided as a Source Data file.

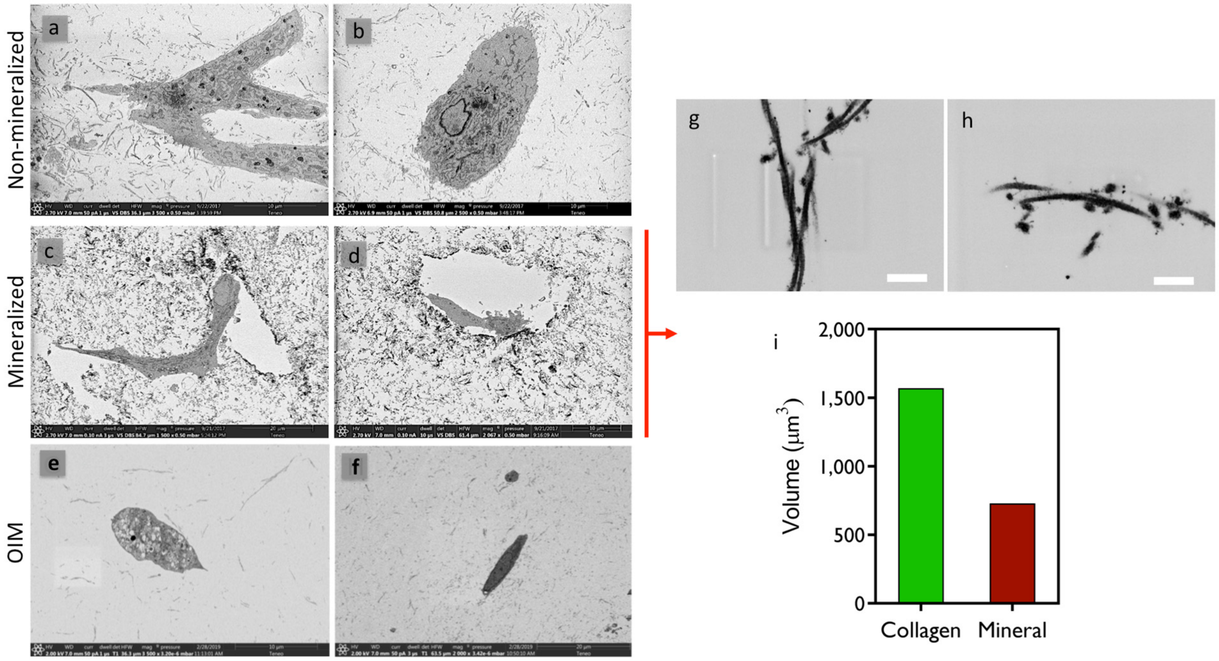


**Supplementary Figure 16:** a-f) Backscattered SEM images acquired in serial block face imaging acquisition mode, from separate samples in non-mineralized, mineralized and OIM-treated groups. The dark contrast areas are due to the backscattered electrons emitted by minerals or denser features (i.e. cells and organelles), whereas the lower contrast areas are non-mineralized collagen or void spaces. Non-mineralized samples lacked any visible contrast (a-b). Mineralized samples had a markedly darker contrast throughout the matrix, and particularly greater mineral formation in the regions bordering the pericellular space, which is consistent the lamina limitans formed around osteocytes in osteonal bone (c-d). Matrix in OIM-treated samples had no visible backscattered contrast (e-f), consistent with lack of mineralization after 7 days in OIM medium. Void spaces around cells in (c-d) are likely artifacts from sectioning/processing. (g-h) Selected samples from the mineralized group were also processed via focused ion beam (FIB) milling and imaged in higher magnification in backscattered mode to illustrate de density of the mineral contracts at the individual fibril level. Scale bar: 500 nm. (N=3) i) Collagen and mineral volume calculated from the 3D reconstructed image of cell-laden mineralized matrix showed that approximately 47% of the collagenous matrix was covered by mineral. For that, serial slices of resin embedded mineralized constructs obtained at 60 nm intervals using serial block face SEM was aligned and processed using AMIRA (Version 5) image analysis and reconstruction software. Subsequently, semi-automated segmentation of mineral and collagen was performed slice-by-slice, followed by volume rendering to compute the 3D volume occupied by the specific features of interest. Source data are provided as a Source Data file.

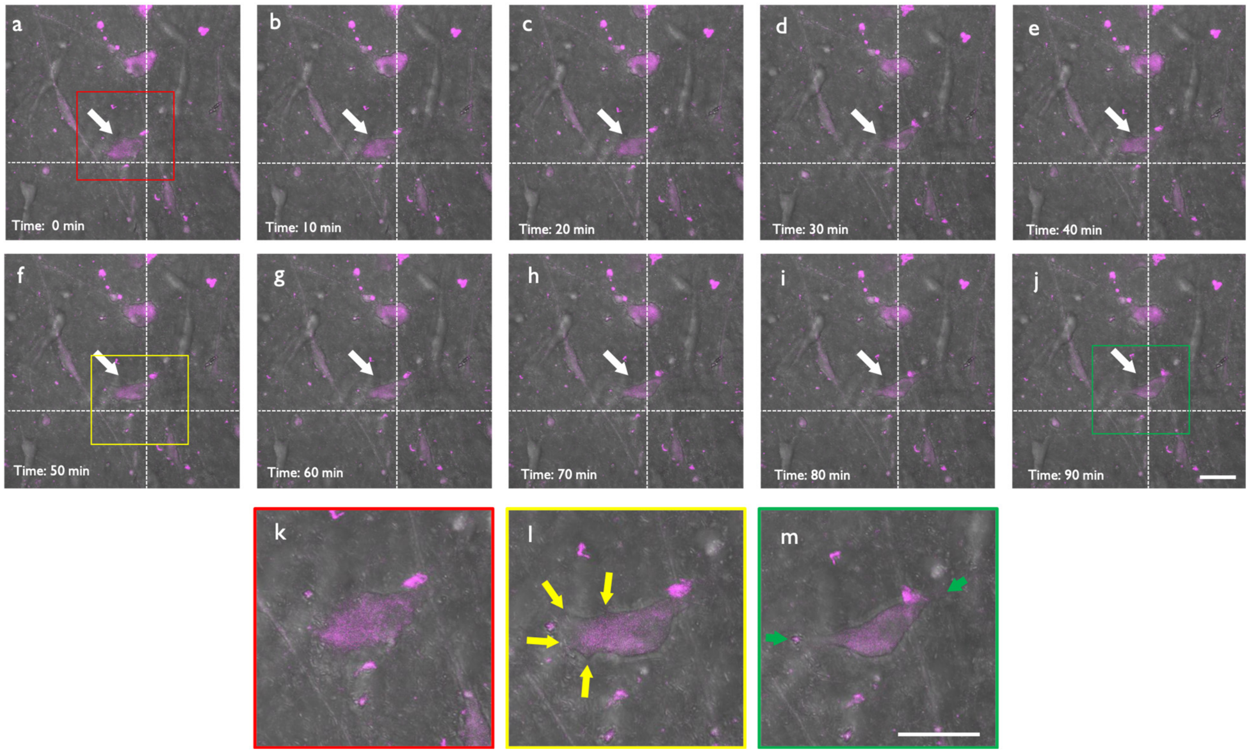


**Supplementary Figure 17:** Time lapse imaging sequence for hMSCs encapsulated in a mineralized collagen hydrogel (N=3) on day 7. Images were acquired at 20X magnification for 90 min at 10-min intervals with Zeiss airyscan 880 microscope and processed with ImageJ software. Cells were stained using a cell tracker (deep red cell tracker, Invitrogen, 1 ug/ml) and images were generated both in fluorescence and transmission modes, and then merged. A single cell is shown with a white arrow to illustrate cell movement within the mineralized matrix. On the image in (a), the right and lower edges of the cell are bordered with a tangential dotted line to mark the starting point of a cell at time point 0; the lines are not moved across the time points for easier reference. The movement of the cell away from the reference lines facilitates visualization of the movement path of the cell from 0 to 90 min (a-j). A zoomed-in view of a single cell at time points 0 (k), 50 (l) and 90 min (m) show the morphological changes to the cell cytoplasm and changes in the formation of cell processes that extend and contract in different directions over time. In (l) multiple narrow processes are shown in a cell (yellow arrow), and (m) after 90 min, these processes have either disappeared or moved to another location (green arrows). A video of the time-lapse images is shown in Supplementary Video 3, and a comparison for cells cultured in the osteoinductive medium is shown in Supplementary Video 4. Scale bar: 50 µm.

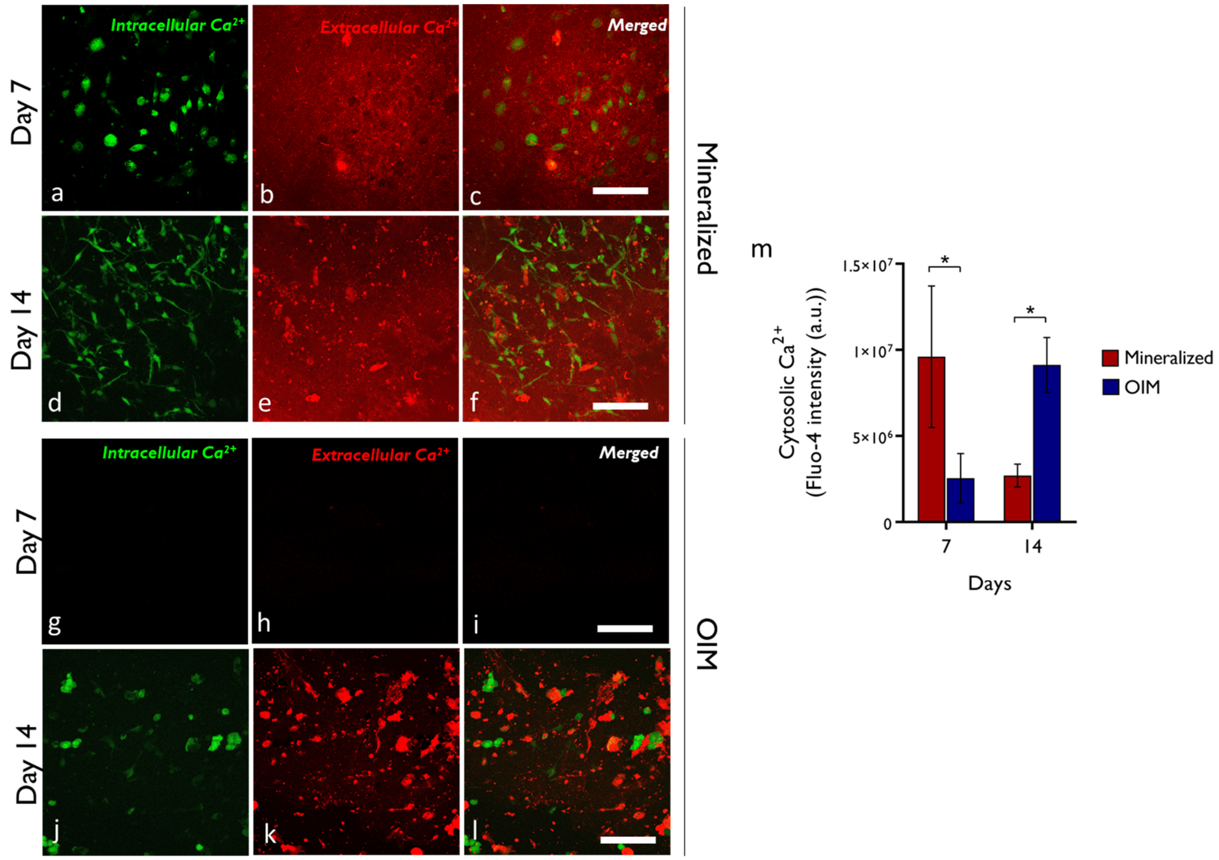


**Supplementary Figure 18:** Dual calcium indicators (cell permeant Fluo-4AM dye (494/506 nm) and Rhod-5N dye (551/576 nm)) were used to distinguish intracellular and extracellular $Ca2^{+}$, respectively. After 7 and 14 days of culture in mineralized and OIM treated collagen matrix, hMSCs were loaded with 5 uM of Fluo-4 AM for 2 hours. Post incubation, excess Fluo-4 AM was rinsed off, followed by loading of 5 uM of Rhod-5N and the samples were subsequently imaged live to visualize the increase in fluorescence intensity upon binding to $Ca2^{+}$ using LSM 880 Laser scanning Confocal microscope (N=3). Cells cultured in mineralized collagen (a-c) had a two-fold increase in cytosolic calcium (green) on day 7, compared to those cultured in OIM (*$p<0.05$). For OIM, these levels were only attained on day 14 (j-l). Extracellular calcium (red) appears to be distributed throughout the matrix since day 7 in mineralized collagen (b-c, e-f), with higher intensity spots surrounding cells on day 14, possibly due to cell-secreted minerals. OIM samples had very little extracellular calcium on day 7 (h-i) and only localized calcium deposits in the pericellular regions on day 14 (k-l). Scale bar: 50 µm. Source data are provided as a Source Data file.

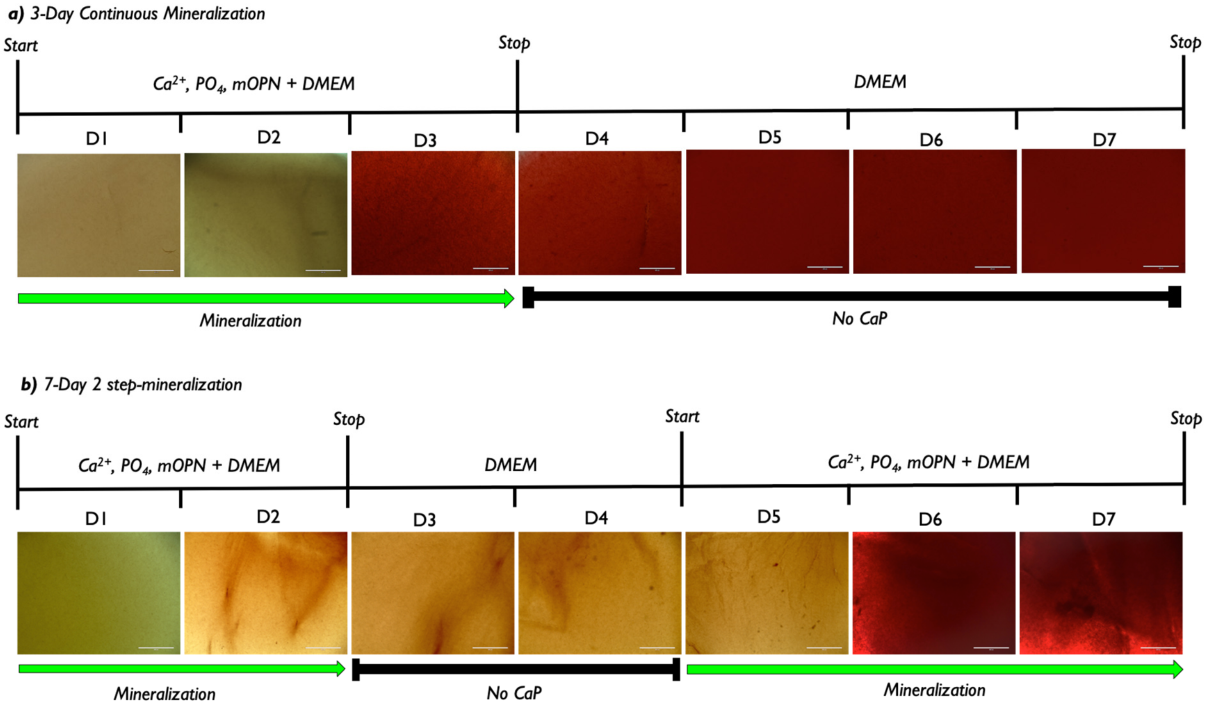


**Supplementary Figure 19:** Alizarin red staining showing the time-controlled mineralization of collagen hydrogel. a) Sample mineralization for 3 continuous days. Samples were then maintained in DMEM medium for the remainder of the experiment. b) Partial calcification of the collagen matrices was induced by exposing the samples to mineralizing medium (cell-culture medium with supersaturated Ca and P ions stabilized with mOPN) for 2 days. Next, the medium was replaced with standard DMEM without additional Ca and P or mOPN, to stop mineralization until day 4. Note the faint red staining till day 5 due to limited mineralization of the matrix. On day 5, samples were cultured with the Ca, P and mOPN rich medium again, thus resuming the mineralization process and completing matrix calcification, as visualized from the intense red staining at later time-points. After each time-point, the samples were fixed, stained with 2% Alizarin red S and imaged under bright field mode using EVOS FL Auto 2 Imaging System (N=6). Scale bar: 400 µm.

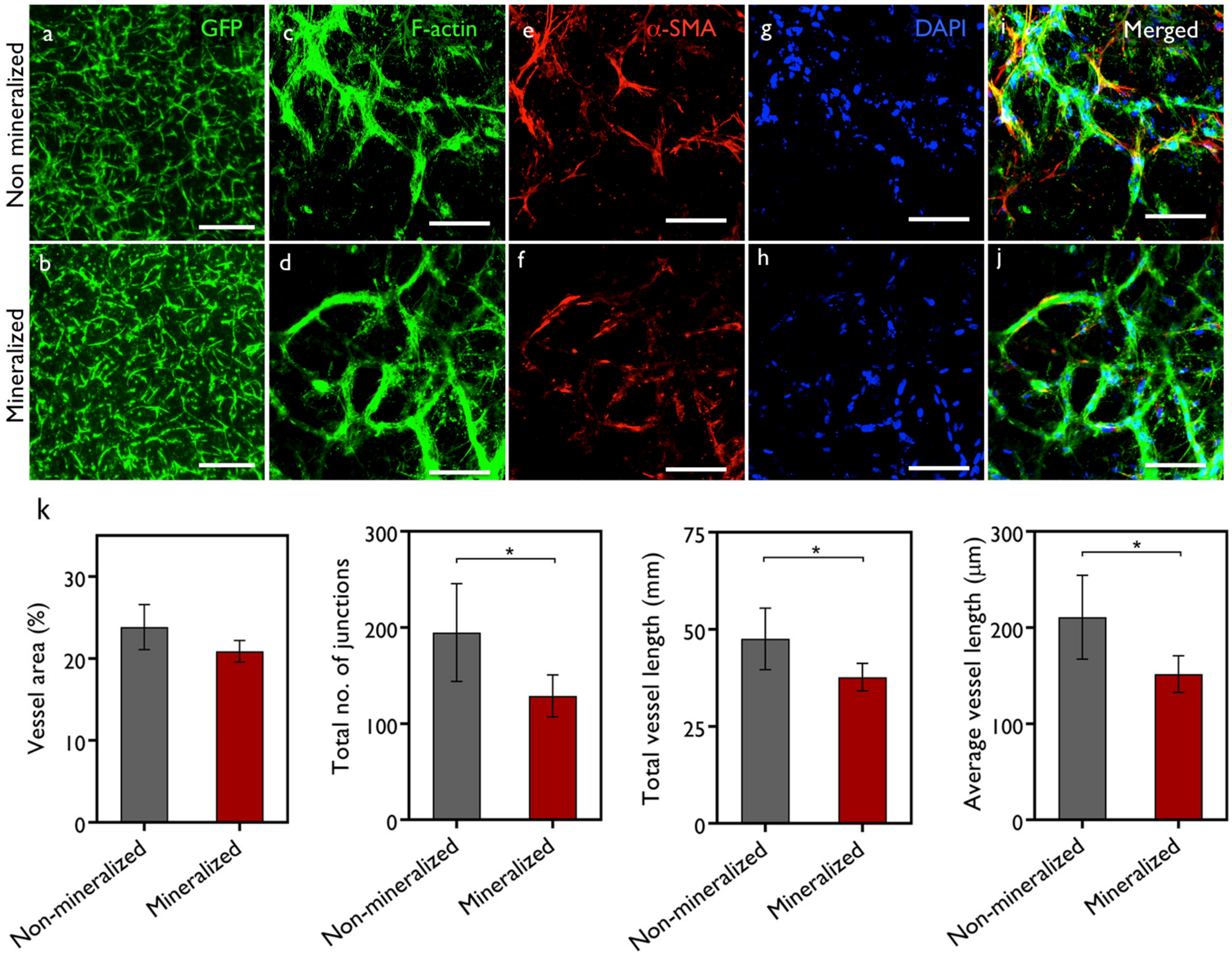


**Supplementary Figure 20:** a-b) Fluorescence image of GFP-expressing hUVECS in co-culture with hMSCs (4:1 ratio) encapsulated in non-mineralized and mineralized hydrogels after 7 days in culture. Dense capillary-like networks are visible after 3 days of matrix mineralization, and mineralization had negligible effects on cell and network morphology. Scale bar: 500 µm. c-j) Representative immunofluorescence images depicting pericyte-supported endothelial network formation in non-mineralized and mineralized constructs. hMSCs grown in close contact with the hUVECs showed high expression for αSMA, a marker indicative of the pericytic differentiation of hMSCs. Scale bar: 100 um. k) Quantification of percentage vessel area, total number of vessel junctions, total vessel length and average vessel length by Angiotool software. A reduction in vasculature formation in-vitro suggest that the mineralization process affected vasculogenesis in-vitro (*p<0.05), despite effective endothelial network formation in both groups, and recovery of vascular density after implantation. Data represented as Mean±SD (N=6). Source data are provided as a Source Data file.

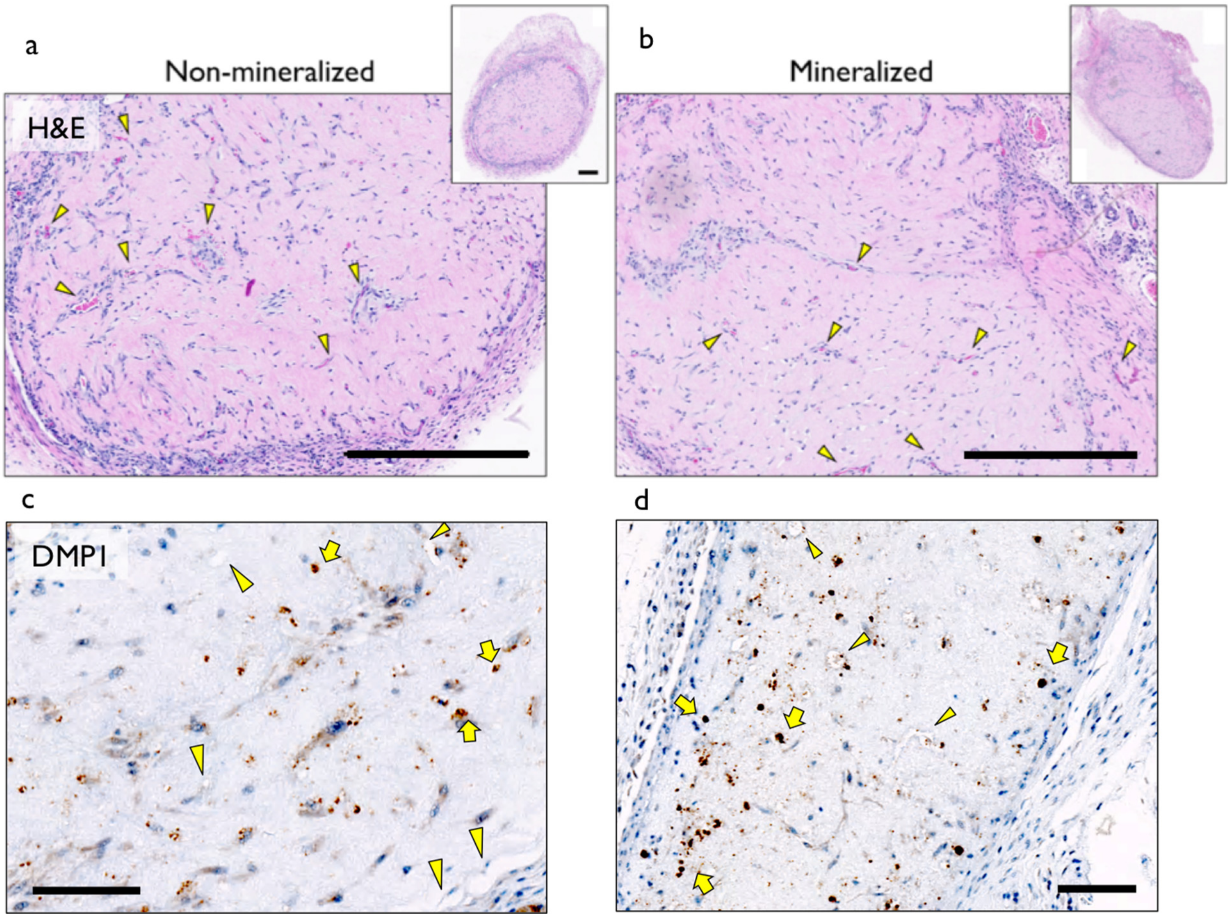


**Supplementary Figure 21**: a) Engraftment of non-mineralized and (b) mineralized hydrogels in-vivo. H&E image showing erythrocyte filled capillaries (yellow arrow head) within the constructs after 3 weeks of implantation in nude mice. Scale bar: 500 µm. c) Expression of DMP1 was visible in (c) non-mineralized and (d) mineralized constructs implanted with pre-formed vasculature after 7 days of culture in-vitro, followed by 7 days in-vivo. DMP1-expressing cells (yellow arrows), which are evident even in mineralized constructs some of which are present near cross-sectioned luminal structures that are consistent with the appearance of vasculature capillaries (yellow arrowheads, unstained), demonstrate osteoblast formation in conjointly with vasculature in these mineralized samples.  Scale bar: 100 µm (N=4).

## Supplementary Table

| Gene code | **Nucleotide Sequence (5′ – 3′)** |
|---|---|
| **ALP** | For: ACATTCCCACGTCTTCACATTT<br>Rev: AGACATTCTCTCGTTCACCGCC |
| **OCN** | For: TGTGAGCTCAATCCGGACTGT<br>Rev: CCGATAGGCCTCCTGAAGC |
| **RUNX2** | For: AGATGATGACACTGCCACCTCTG<br>Rev: GGGATGAAATGCTTGGGAACT |
| **DMP1** | For: AGAAGCGAGCTTGATGACAACAA<br>Rev: TGGACTCACTGCTGGGACCATCTAC |
| **PDPN** | For: TTACTAGCCATCGGCTTCATT<br>Rev: GGCGAGTACCTTCCCGACAT |
| **GAPDH** | For: AACAGCGACACCCACTCCTC<br>Rev: CATACCAGGAAATGAGCTTGACAA |

**Supplementary Table 1:** Primer sequences for real time qPCR
